# Age-related differences in monocyte DNA methylation and immune function in healthy Kenyan adults and children

**DOI:** 10.1186/s12979-021-00223-2

**Published:** 2021-03-08

**Authors:** Katherine R. Dobbs, Paula Embury, Emmily Koech, Sidney Ogolla, Stephen Munga, James W. Kazura, Arlene E. Dent

**Affiliations:** 1 Center for Global Health and Diseases, Case Western Reserve University, 10900 Euclid Avenue LC: 4983, Cleveland, OH 44106 USA; 2 Division of Pediatric Infectious Diseases, University Hospitals Rainbow Babies and Children’s Hospital, Cleveland, OH USA; 3 Centre for Global Health Research, Kenya Medical Research Institute, Kisumu, Kenya

**Keywords:** Monocyte, DNA methylation, Epigenetic, Ageing, Aging, Innate immune

## Abstract

**Background:**

Age-related changes in adaptive and innate immune cells have been associated with a decline in effective immunity and chronic, low-grade inflammation. Epigenetic, transcriptional, and functional changes in monocytes occur with aging, though most studies to date have focused on differences between young adults and the elderly in populations with European ancestry; few data exist regarding changes that occur in circulating monocytes during the first few decades of life or in African populations. We analyzed DNA methylation profiles, cytokine production, and inflammatory gene expression profiles in monocytes from young adults and children from western Kenya.

**Results:**

We identified several hypo- and hyper-methylated CpG sites in monocytes from Kenyan young adults vs. children that replicated findings in the current literature of differential DNA methylation in monocytes from elderly persons vs. young adults across diverse populations. Differentially methylated CpG sites were also noted in gene regions important to inflammation and innate immune responses. Monocytes from Kenyan young adults vs. children displayed increased production of IL-8, IL-10, and IL-12p70 in response to TLR4 and TLR2/1 stimulation as well as distinct inflammatory gene expression profiles.

**Conclusions:**

These findings complement previous reports of age-related methylation changes in isolated monocytes and provide novel insights into the role of age-associated changes in innate immune functions.

**Supplementary Information:**

The online version contains supplementary material available at 10.1186/s12979-021-00223-2.

## Background

Development of the human immune system is a highly dynamic process influenced by genetic and environmental factors, including early-life exposures which can impact long-term risk for immune-mediated diseases [1–3]. Susceptibility to infection varies according to age, with newborns and the elderly generally at the highest risk [4, 5]. The effects of an aging immune system are well characterized in the elderly (aged 65 years or older), which include weakened responses to pathogens and vaccines (immunosenescence) and chronic, low-grade inflammation (inflamm-aging) [6–8].

Monocytes are innate immune cells important to phagocytosis, inflammatory cytokine production, antigen presentation, and tissue repair, and play a central role in dysregulated innate immune responses that characterize inflamm-aging [9]. Age-related changes in monocytes from older vs. young adults include several altered gene expression programs, including those linked to metabolic homeostasis, antigen presentation, authophagy, and protein synthesis [10–13]. Cytokine production in response to pattern recognition receptor (PRR) engagement is dysregulated in monocytes from older vs. young adults. Studies have shown increased TNF and decreased CCL20, IFN-γ, and IL-1β production in response to LPS treatment in elderly monocytes [11, 14, 15]. Additional studies have shown decreased type I IFN production after stimulation with 5’pppRNA and influenza [11, 16]; decreased IL-1β and IFN-γ with CL097 stimulation [11]; and decreased IL-6 and TNF with Pam3Cys stimulation [17]. Other age-associated changes in elderly monocytes include decreased phagocytosis and altered cellular metabolism, including reduced mitochondrial capacity and increased glucose consumption [10, 14].

The development, differentiation, and functions of immune cells are regulated by dynamic epigenetic modifications, including histone modifications and DNA methylation of cytosines in CpG dinucleotides [18]. DNA methylation patterns are influenced by genetic sequence and a host of environmental factors, including nutrition and infectious exposures [19]. Age-related DNA methylation patterns have been well described in multiple tissue types in adults, and methylation patterns at age-associated CpG sites have been analyzed to produce highly accurate and precise estimates of chronological age in older age groups [20, 21]. DNA methylation changes associated with aging include genomic regions with increased methylation with older age (hyper-methylation) as well as regions with decreased methylation with older age (hypo-methylation) [13, 22–25]. Previous studies of both children and adults have shown that CpG sites that are hypo-methylated with age are located in CpG island shores and outside of CpG islands [24, 26, 27]. Several studies in adult populations have shown that CpG sites that are hyper-methylated with age are enriched in CpG islands [13, 26–28], although a study in children did not find enrichment of age-associated hyper-methylated sites within CpG islands [24]. There are limited data available regarding age-associated DNA methylation changes in pediatric populations, although existing evidence suggests that age-related methylation changes in peripheral blood occur more rapidly in childhood than adulthood, and that changes in many age-associated CpG sites follow a logarithmic rather than linear lifelong trend [24].

Most studies of age-related DNA methylation changes have used whole blood or other mixed cell samples, and few have studied methylation changes in purified monocyte samples [10, 12, 13, 29–31]. Studies of age-associated methylation patterns in purified T cells and monocytes from the same individuals showed distinct methylation changes in T cells vs. monocytes and emphasize the importance of studying isolated cell populations [12, 13]. Additionally, it is important to take into account the influence that genetic factors and gene-environment interactions have on the aging epigenome, yet the majority of studies on age-related methylation markers have been performed in populations with European ancestry, and few have been performed in African populations [32, 33]. Here, we aimed to analyze DNA methylation profiles and innate immune phenotypes and functions of purified monocytes from healthy individuals living in western Kenya, comparing children aged 1–9 years to adults aged 19–35 years. Participants for this study were enrolled in a larger observational cohort study of naturally acquired immunity to malaria [34, 35]. Malaria transmission in this area is perennially high, and malaria-related morbidity and mortality is highest among children under 5 years [36, 37]. By young adulthood, individuals living in this region develop clinical immunity in which they are protected against severe malarial disease but occasionally have episodes of uncomplicated malaria as well as subclinical parasitemia. Findings from this study reveal age-related differences in monocyte DNA methylation, innate immune responses, and inflammatory gene expression patterns that provide insights into differential age-related risks for both infectious and non-infectious diseases in populations living in malaria-endemic areas. Monocytes from healthy malaria-naïve adults from the United States were examined as historic controls to highlight the important considerations of genetic and ecologic diversity in studies of the developing and aging immune system.

## Results

### Age-related differences in monocyte DNA methylation in Kenyan adults vs. children

We analyzed genome-wide DNA methylation profiles in negatively selected monocytes from 8 healthy children (aged 3.6–9.3 years) and 8 healthy young adults (aged 26–30 years) from western Kenya (Additional file 1: Table S1). Methylation profiles were determined using the Infinium MethylationEPIC bead array, which interrogates methylation at > 850,000 sites across the genome at single-nucleotide resolution. In the analysis of differentially methylated positions (DMPs) and differentially methylated regions (DMRs), we filtered out probe sites located at known single nucleotide polymorphisms (SNPs) and those on the X and Y chromosomes. Eighteen percent of children in the overall cohort had asymptomatic Pf parasitemia (a common finding in this malaria endemic region), so we included sex and asymptomatic Pf parasitemia status as covariates to adjust for potential confounding effects.

**Fig. 1:**
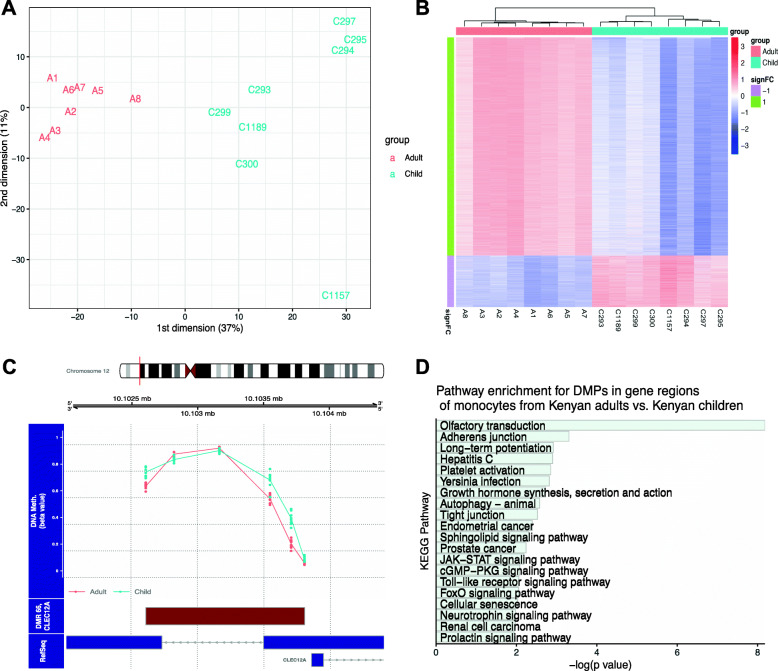
Age-related differences in monocyte DNA methylation in Kenyan adults and children. Monocytes were negatively selected from fresh venous blood samples from healthy Kenyan children (*n* = 8) and adults (*n* = 8). **a** Principal components analysis (PCA) plot of DNA methylation results for Kenyan adult (red) and child (blue) monocytes. **b** Heatmap of DNA methylation results showing differentially methylated positions between Kenyan adult and child monocytes, includes all CpG probe sites meeting differential methylation criteria (absolute beta value difference > 0.15, *p* < 0.01, and FDR-adjusted *p* < 0.05). **c** Plot of differentially methylated region in the *CLEC12A* gene locus, showing beta values for Kenyan adult (red) and child (blue) monocytes at 6 CpG probe sites within the region. **d** KEGG pathway enrichment analysis of genes associated with differentially methylated positions in monocytes from Kenyan adults vs. children, showing the most significantly enriched pathways

Principal components analysis (PCA) showed that principal component 1 (PC1) accounted for 37% of variance in the data and was significantly associated with age group (*p* = 0.0008) (Fig. 1a). Using a threshold for absolute beta value difference of 0.15, we identified 14,259 differentially methylated CpG sites, with 11,535 sites hyper-methylated in adults vs. children and 2724 hypo-methylated in adults vs. children (*P* < 0.01, FDR-adjusted *P* < 0.05) (Fig. 1b; Additional file 2). Table 1 highlights DMPs in our study that replicate findings in the literature of age-related DMPs in isolated monocytes, DMPs that are relevant to monocyte immune functions, and the top five most significant DMPs.

**Table 1:**
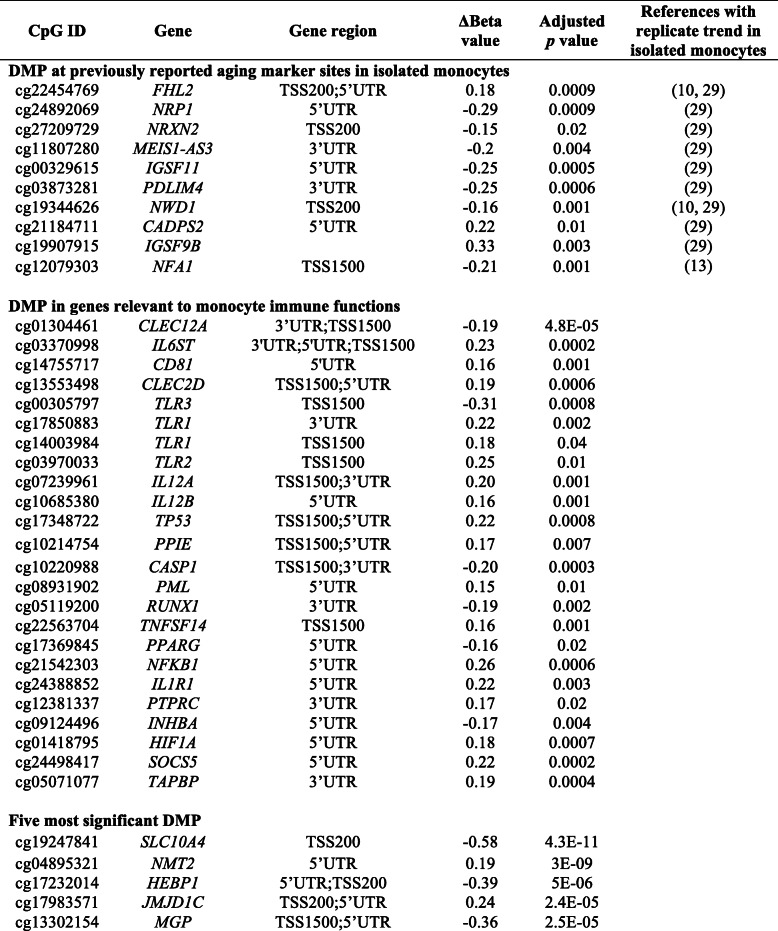
Differentially methylated positions in monocytes from Kenyan adults vs. children

We found DMPs at CpG sites previously reported to have age-associated differential methylation in isolated monocytes [10, 13, 29] as well as whole blood [20, 21, 33] (Table 1; Additional file 2). For example, we found hyper-methylation in adults vs. children at the site cg22454769 in the *FHL2* locus, and similar findings of hyper-methylation with older age have been reported in whole blood samples [20, 21] as well as in studies of isolated monocytes by Tserel et al. [29] and Saare et al. [10]. Several DMPs in our study showed similar patterns in adults vs. children as those found by Tserel et al. in isolated monocytes from old vs. young adults [29], including hypo-methylation at sites cg24892069 in the *NRP1* locus and cg27209729 in the *NRXN2* locus. We also found hypo-methylation at the site cg18334392 in the *SNORD123* locus (Δbeta = − 0.2, adj. *p* = 0.03), and both DMPs in the *NRXN2* and *SNORD123* loci were previously identified in an epigenome wide association study (EWAS) meta-analysis (using samples from multiple tissue types) of an African hunter-gatherer group and also hypo-methylated with older age [33]. Well-known CpG sites in the *ELOVL2* promoter are associated with hyper-methylation with increasing age [20, 38], and this correlation has been consistent across tissue types [39] and populations [33]. In our study, three CpG sites in *ELOVL2* followed this trend with hyper-methylation in adults vs. children but did not meet our 0.15 beta-difference threshold (cg16867657, Δbeta = 0.12, adj. *p* = 0.06; cg07901130, Δbeta = 0.14, adj. *p* = 0.01; cg08683008, Δbeta = 0.06, adj. *p* = 0.01). We found that CpG probe sites that were hypo-methylated in adults vs. children were more likely to be located in CpG shores (22.2% of hypo-methylated sites were in CpG shores, compared to assay coverage of 17.8%, *p* < 0.0001), while CpG probe sites that were hyper-methylated in adults vs. children were not enriched in CpG islands.

Several DMPs were contained in gene regions important to monocyte inflammatory and immune responses (Table 1; Additional file 2). These include CpG sites in genes for PRRs (*CLEC12A, CLEC2D, TLR2, TLR1, TLR3*), inflammasome and cytokine signaling (*CASP1, IL6ST, IL12A, IL12B, IL1R1*), and downstream signaling molecules and transcription factors (*TP53, PPARG, NFKB1, PTPRC, HIF1A*). In addition, several DMRs (regions containing multiple DMPs) were in gene regions relevant to immune function, including *CLEC12A* (6 CpG sites, combined *p* = 1.3E-07) (Fig. 1c), *IL6ST* (3 CpGs, *p* = 1.8E-08), *TLR1* (9 CpGs, *p* = 0.01), *TLR3* (8 CpGs, *p* = 0.0008), and *IL12A* (15 CpGs, *p* = 0.02) (Additional file 3).

We performed enrichment analysis of DMPs in gene regions of monocytes from Kenyan adults vs. children for Kyoto Encyclopedia of Genes and Genomes (KEGG) pathways, which was notable for enrichment in several important signaling pathways (including Sphingolipid, JAK-STAT, cGMP-PKG, Toll-like receptor, and FoxO signaling pathways), “Cellular senescence,” and several malignancy-related pathways (Fig. 1d; Additional file 4).

### Monocyte subset proportions are similar in Kenyan adults and children

Circulating monocytes are a heterogeneous population that are classified into three subsets according to CD14 and CD16 expression (classical CD14^++^CD16^−^, intermediate CD14^++^CD16^+^, and nonclassical CD14^+^CD16^++^). The subsets have characteristic phenotypic, functional, transcriptomic, and epigenetic profiles [40, 41], including subset-specific miRNA expression [42] and DNA methylation profiles linked to distinct immunological processes [43]. Several studies have shown an increase in circulating CD16^+^ subsets in elderly vs. young adult monocytes [14, 15, 17], though others have shown no difference in proportions of subsets between elderly and young adults [11]. To determine if there were baseline differences in proportions of monocytes subsets in Kenyan young adults vs. children, we performed flow cytometry to calculate percentages of classical, intermediate, and nonclassical subsets in PBMC samples from 17 healthy children (aged 1.2–9.6 years) and 14 healthy adults (aged 18–35 years). We found no differences between the two groups in proportions of the three subsets (Additional file 1: Fig. S1).

### Increased TLR4 and TLR2/1 responsiveness in monocytes from Kenyan adults vs. children

**Fig. 2:**
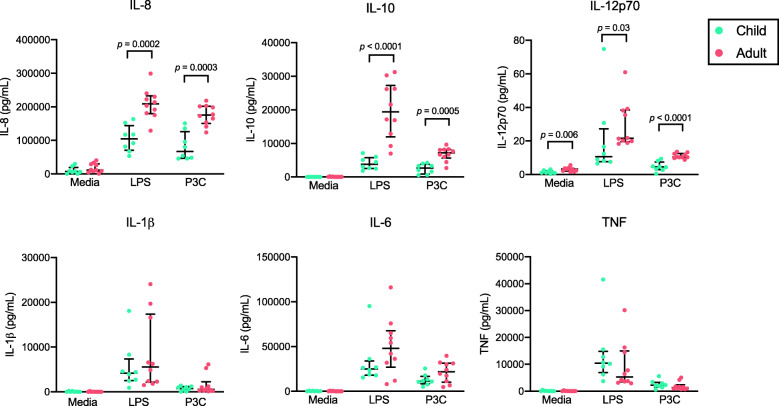
Monocyte responsiveness to TLR4 and TLR2/1 stimulation in Kenyan adults and children. Monocytes were negatively selected from fresh venous blood samples from healthy Kenyan children (*n* = 8) and adults (*n* = 10). Cells were cultured for 18 h with media alone, 10 ng/ml LPS, or 100 ng/ml Pam3CSK4 (P3C), and cytokine concentrations were measured in culture supernatants. Mann Whitney U test was used to compare the groups. Data are shown as medians with interquartile ranges

We analyzed monocyte cytokine production using negatively isolated monocytes from fresh venous blood samples obtained from 8 healthy children (aged 3.9–9.8 years) and 10 healthy adults (aged 26–30 years). Cells were cultured 18 h, and constitutive cytokine production (media alone) was compared to stimulation with a TLR4 agonist (LPS 10 ng/ml) and a TLR2/1 agonist (Pam3CSK4 [P3C] 100 ng/ml). Monocytes from adults showed increased production of IL-8, IL-10, and IL-12p70 in response to both TLR4 and TLR2/1 stimulation compared to monocytes from children (Fig. 2). Constitutive production of IL-12p70 was slightly higher in adult monocytes (median 3.2 pg/ml) vs. child monocytes (median 1.1 pg/ml) (*p* = 0.006). Production of IL-1β, IL-6, and TNF in response to TLR4 and TLR2/1 agonists was robust and equal in monocytes from Kenyan adults and children (Fig. 2).

### Differential inflammatory gene expression in monocytes from Kenyan adults vs. children

**Fig. 3:**
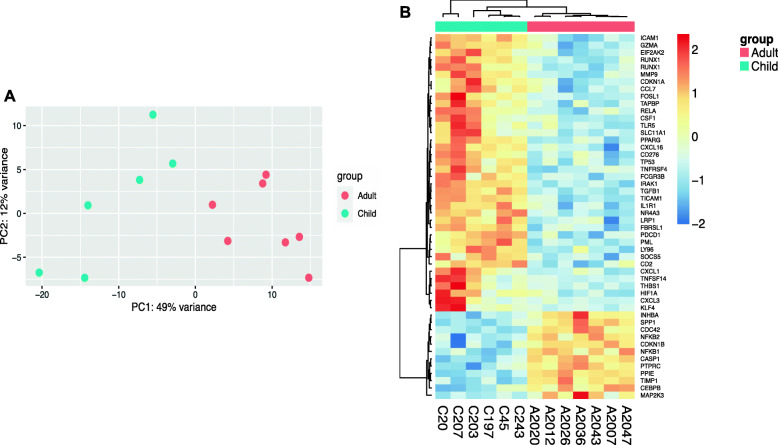
Monocyte inflammatory gene expression profiles in Kenyan adults and children. Targeted digital RNA sequencing was performed on monocytes isolated from cryopreserved PBMCs via negative selection. A customized panel targeted 508 genes important in inflammation and immunity (QIAseq Human Inflammation and Immunity Transcriptome Panel, Qiagen). **a** Principal components analysis (PCA) plot of targeted digital RNA sequencing results for Kenyan adult (red) and child (blue) monocytes. **b** Heatmap of the top 50 most significantly differentially expressed genes between Kenyan adult and child monocytes

We examined inflammatory gene expression profiles of negatively isolated monocytes from 6 healthy Kenyan children (aged 3.3–4.9 years) and 7 healthy Kenyan adults (aged 28–33 years) using a targeted digital RNA sequencing panel, which measured expression of 508 genes involved in inflammation and immunity. PCA analysis showed separation of the two groups along the first principal component, which explained 49% of variance in the data (Fig. 3a). Of the 508 genes included in the panel, 178 were differentially expressed between adult and child monocytes (73 genes had increased expression in adult vs. child monocytes, 105 genes had decreased expression in adult vs. child monocytes, adj. *p* < 0.05) (Additional file 5). The top 50 differentially expressed genes are shown in Fig. 3b and include several of the genes in which differential methylation was observed, such as *CASP1, IL1R1, TP53, PPARG, NFKB1, PTPRC,* and *HIF1A.* Of the 508 inflammation-associated genes analyzed, 135 (26.6%) had differential methylation observed in promoter or gene body regions in Kenyan adults vs. children. The three most significantly enriched KEGG pathways for differential gene expression included “Cytokine-cytokine receptor interaction” (45 genes in list, FDR *p* = 1.1E-45), “Toll-like receptor signaling pathway” (27 genes in list, FDR *p* = 2.1E-32), and “IL-17 signaling pathway” (25 genes in list, FDR *p* = 1.5E-30). Of note, monocyte samples for DNA methylation and gene expression analyses were obtained from different individuals, so direct correlation between differential methylation and mRNA levels could not be performed.

### Differences in DNA methylation, immune phenotypes, and gene expression in monocytes from Kenyan adults vs. US adults

To examine the potential influence of differences in geographic location and ancestry on age-related differences in monocytes, we also compared monocyte DNA methylation, subset proportions, TLR responses, and inflammatory gene expression profiles of healthy Kenyan adults (median age 28.5 years, range 18–35) to healthy adults from the United States (median age 29 years, range 24–69) (Additional file 1: Table S1).

**Fig. 4:**
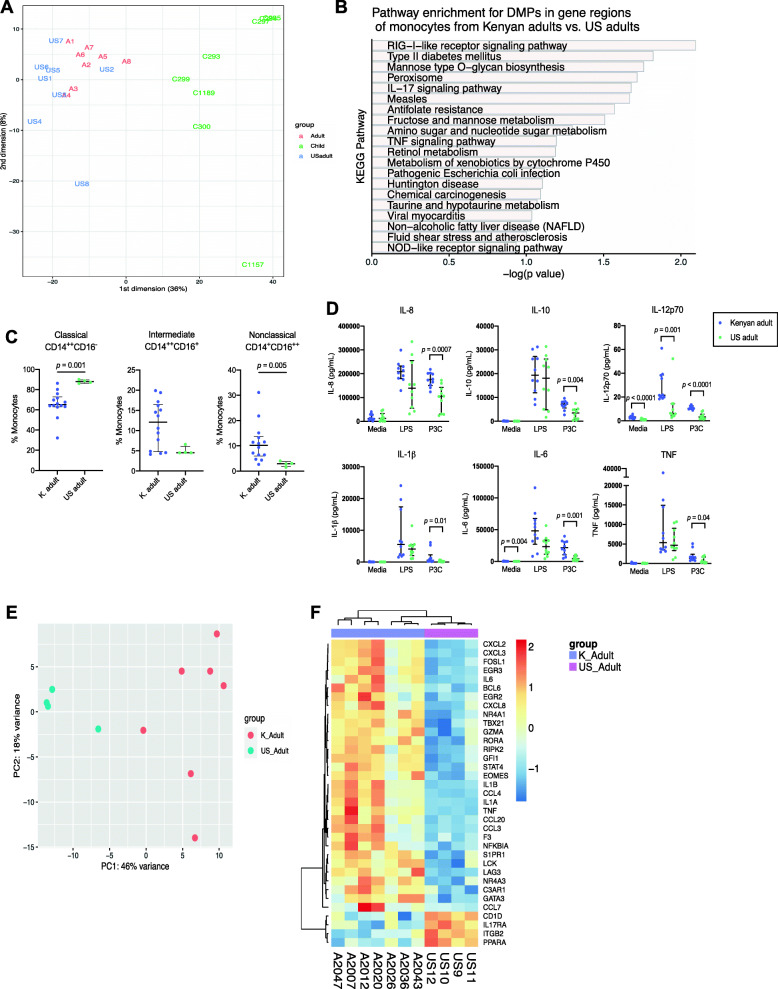
Epigenetic, phenotypic, functional, and transcriptional differences in monocytes from Kenyan adults vs. US adults. **a** Principal components analysis (PCA) plot of DNA methylation results for Kenyan adult (red; *n* = 8), Kenyan child (green; *n* = 8), and US adult (blue; *n* = 8) monocytes. **b** KEGG pathway enrichment analysis of genes associated with differentially methylated positions in monocytes from Kenyan adults vs. US adults, showing the most significantly enriched pathways. **c** Proportions of all circulating monocytes that are classical, intermediate, and nonclassical for PBMC samples from Kenyan adults (*n* = 14) and US adults (*n* = 4). Mann Whitney U test was used to compare the groups. **d** Monocyte cytokine production in response to 18 h of TLR4 (LPS 10 ng/ml) and TLR2/1 (P3C 100 ng/ml) stimulation in Kenyan adults (*n* = 10) and US adults (*n* = 14). Monocytes were negatively selected from fresh venous blood samples. Mann Whitney U test was used to compare the groups. Data are shown as medians with interquartile ranges. **e** PCA plot of targeted digital RNA sequencing results for Kenyan adult (red; *n* = 7) and US adult (blue; *n* = 4) monocytes. **f** Heatmap of the top 35 most significantly differentially expressed genes between Kenyan adult (blue) and US adult (purple) monocytes

A PCA plot of monocyte DNA methylation profiles of Kenyan children, Kenyan adults, and US adults showed separation of the Kenyan child group (*n* = 8) from both groups of adults (*n* = 8 Kenyan adults and *n* = 8 US adults) along PC1 (Fig. 4a). There were a far greater number of DMPs between Kenyan children and US adults (20,919 DMPs) than between Kenyan adults and US adults (288 DMPs) (Additional files 6 and 7). Differential methylation at known aging marker sites was consistent when comparing Kenyan children to both Kenyan adults and US adults. For example, methylation at cg22454769 in *FHL2* was hyper-methylated in both Kenyan and US adult groups compared to Kenyan children (average beta values 0.49, 0.49, and 0.32, respectively, adj. *p* = 9.3E-05), as was methylation at cg16867657 in *ELOVL2* (average beta values 0.44, 0.51, and 0.32, respectively, adj. *p* = 0.02). KEGG pathway enrichment analysis of DMPs in gene regions of monocytes from Kenyan adults vs. US adults was notable for enrichment in several pathways related to immune function and metabolism (such as “RIG-I-like receptor signaling pathway,” “Type II diabetes mellitus,” and “Mannose type O-glycan biosynthesis”) (Fig. 4b; Additional file 8). These data suggest that age may be a larger driver of differential methylation in monocytes than geographic location or ancestry, though the differences in methylation profiles between Kenyan adults vs. US adults may be explained in part by differences in genetic background as well as cumulative environmental exposures, such as malaria.

We compared proportions of circulating classical, intermediate, and nonclassical monocyte subsets in 14 Kenyan adults and 4 US adults, and found that Kenyan adults had overall greater variability, higher proportions of the CD14^+^CD16^++^ nonclassical subset, and lower proportions of the CD^++^CD16^−^ classical subset (Fig. 4c). We then compared monocyte cytokine production in response to TLR2/1 and TLR4 stimulation in 10 Kenyan adults and 10 US adults. Monocytes from healthy Kenyan adults showed consistently higher TLR2/1 responses compared to monocytes from healthy US adults, with increased production of IL-8, IL-10, IL-12p70, IL-1β, IL-6, and TNF after stimulation with P3C (Fig. 4d). In addition, monocytes from Kenyan adults vs. US adults produced higher levels of IL-12p70 in response to TLR4 stimulation with LPS (Fig. 4d).

Using the targeted digital RNA sequencing panel of 508 genes involved in inflammation and immunity, we compared gene expression profiles of monocytes from 7 healthy Kenyan adults to 4 healthy US adults. PCA analysis showed separation of the two groups along the first principal component, which explained 46% of variance in the data (Fig. 4e). Of the 508 genes included in the panel, 121 were differentially expressed between Kenyan adult vs. US adult monocytes (80 genes had increased expression in Kenyan adult vs. US adult monocytes, 41 genes had decreased expression in Kenyan adult vs. US adult monocytes, adj. *p* < 0.05) (Additional file 9). The top 35 differentially expressed genes are shown in the heatmap in Fig. 4f, including upregulation of several important cytokines, chemokines, and transcription factors. Of the 508 inflammation-associated genes analyzed, five (0.98%) had differential methylation observed in promoter or gene body regions in Kenyan adults vs. US adults. The three most significantly enriched KEGG pathways for differential gene expression included “Cytokine-cytokine receptor interaction” (32 genes in list, FDR *p* = 4.8E-31), “Hematopoietic cell lineage” (19 genes in list, FDR *p* = 3.4E-23), and “IL-17 signaling pathway” (17 genes in list, FDR *p* = 2.7E-20). Of note, the 17th most significantly enriched KEGG pathway was “Malaria” (FDR *p* = 4.6E-13) and included differential expression of the following genes in that pathway: *CXCL8, IL6, CCL2, IL1A, IL1B, TNF, IFNG, VCAM1, ICAM1, CD40,* and *LRP1.* Taken together these data on monocyte subset proportions, TLR responses, and gene expression profiles suggest a hyper-inflammatory state in Kenyan adult monocytes that is distinct from both Kenyan child monocytes as well as US adult monocytes, likely secondary to complex gene-environment interactions and their influence on the aging epigenome.

We examined the overlap in DMPs and differentially expressed genes between Kenyan adults vs. Kenyan children and Kenyan adults vs. US adults. We found 12 CpG sites that were differentially methylated in monocytes from Kenyan adults compared to both Kenyan children and US adults. There were 62 genes in the panel of 508 inflammatory genes that were differentially expressed in Kenyan adults compared to the two other groups. Of these 62 genes, 12 were higher in Kenyan adults and 8 were lower in Kenyan adults compared to both Kenyan children and US adults. The remaining 42 genes were expressed at an intermediate level in Kenyan adults compared to Kenyan children and US adults. One of the 12 overlapping DMPs (cg23061725, located in the 5’UTR of *CASP8;* average beta values 0.57 in Kenyan adults, 0.34 in Kenyan children, and 0.36 in US adults) was noted alongside altered monocyte *CASP8* gene expression among the three groups (average normalized expression values 286 in Kenyan adults, 181 in Kenyan children, and 495 in US adults). *CASP8* encodes Caspase-8, a cysteine-aspartate protease involved in several cellular processes, including programmed cell death, autophagy, and inflammasome activation [44].

## Discussion

In this study, we investigated genome-wide DNA methylation patterns of purified monocytes obtained from children and young adults living in a malaria-endemic area of Kenya. Our findings replicate several of the strongest markers for age-related changes in DNA methylation and identify differentially methylated CpG sites at gene loci relevant to innate immune responses. We also found that while monocyte subset proportions were similar between young adults and children, monocyte cytokine production in response to TLR agonists was increased in adults compared to children, and adult monocytes had a distinct upregulated inflammatory gene expression profile.

Several of the age-related changes in DNA methylation in our study occur at CpG sites that are part of the “epigenetic clock” and correlate with chronological aging across tissue and cell types, and have the potential to be used to predict disease states when this correlation is altered [20, 21]. In older adults, patterns for age-associated methylation changes at many CpG sites appear to follow a linear trend [21], however a meta-analysis of age-related DNA methylation changes in pediatric and adult cohorts revealed that changes in methylation occur at a three- to four-fold higher rate in children than in adults and that lifelong trends for most age-associated CpG sites are best modelled by a logarithmic relationship between beta value and age [24]. A similar finding was reported in a study that included Central African children, in which CpG sites that were hypo-methylated with age were better modeled by a function of logarithmic age [33]. Our analyses were limited to comparisons between children and young adults. Important areas for future study include further defining the relationship between DNA methylation and age across the lifespan.

The relationship between DNA methylation and gene expression is complex [45, 46]. Limitations of our study include small sample size, measurement of monocyte gene expression using a targeted panel rather than the entire transcriptome, and that the samples used for gene expression and DNA methylation measurements were not from the same individual. Reynolds et al. integrated DNA methylation and transcriptome profiles of CD14^+^ monocytes (sample size > 1000) and found that 4.7% of age-associated differentially methylated sites correlated with *cis*-gene expression changes [13]. With a smaller sample size, Saare et al. examined the effect of age on purified monocyte DNA methylation, gene expression, and metabolic functions and found a weak correlation between DNA methylation and gene expression [10]. The functional consequences of age-associated differential DNA methylation and transcription have yet to be elucidated. Saare et al. found age-related differences in cellular metabolic fitness [10], which could plausibly underlie age-related impairment in monocyte functions such as phagocytosis, antigen presentation, and cytokine production [11, 47, 48]. Further research is also needed to determine the transcriptional and epigenetic changes that regulate monocyte differentiation and how differences among the monocyte subsets relate to both age and immunity.

Studies of aging monocytes have shown generally weakened PRR responses in elderly vs. young adults [11, 14–17]. In this study, monocyte cytokine production in response to TLR stimulation was increased in Kenyan young adults compared to children. These differences among age groups may reflect continual epigenetic changes that occur in response to a lifetime of environmental exposures, including infections such as malaria. The relationship between altered DNA methylation and functional changes in monocytes was recently explored in the context of bacterial sepsis [49]. Monocytes from patients with sepsis had altered DNA methylation profiles compared to controls, with several hyper- and hypo-methylated CpG sites in monocytes from patients with sepsis correlating with increased constitutive production of IL-10 and IL-6. In vitro models of LPS-induced monocyte tolerance revealed changes in DNA methylation that reflected changes observed in monocytes from septic patients [49]. In another study of the role of DNA methylation in the regulation of innate immune responses to bacterial infection, Pacis et al. found that monocyte-derived dendritic cell exposure to *Mycobacterium tuberculosis* led to transcriptional activation and downstream active demethylation at thousands of CpG sites [50, 51]. The potential regulatory role that DNA methylation may play in innate immune memory and responses to subsequent stimuli has yet to be determined [52–55].

Epigenetic, transcriptional, and functional changes that occur in innate immune cells during the first few decades of life are likely to have a substantial impact on immunity to pathogens that disproportionately affect young children, most notably malaria. The importance of intrinsic age-related differences in immune responses to malaria infection was highlighted in studies of a migrant population of malaria-naïve children and adults who moved from Java, where malaria was rare, to a malaria-holoendemic area of Indonesian Papua [56–58]. Compared to children, newly arrived adults were much more likely to suffer from severe malaria and require evacuation to a hospital for clinical support [56, 57], yet adults appeared to develop clinical immunity more quickly than did children [58]. Further study of age-related differences in monocyte responses to malaria could provide new insights into disease pathogenesis and the acquisition of clinical immunity.

## Conclusions

In summary, we identified DNA methylation patterns in isolated monocytes from Kenyan young adults that include known markers of aging as well as changes in gene loci important to innate immune and inflammatory responses. Monocytes from this cohort of young adults and children also show differences in TLR responses and inflammatory gene expression profiles. These results will inform future studies of the functional significance of altered monocyte DNA methylation in the aging process and immune system development.

## Methods

### Study site and study participants

Participants in this study were enrolled in an observational cohort study of naturally acquired immunity to malaria conducted at the Chulaimbo Sub-County Hospital in Kisumu County, Kenya. Enrollment and sample collection were conducted between June 2013 and April 2016. The study population were of Luo ethnicity. Malaria transmission in this area is perennially high with peaks coinciding with seasonal rains [36], and *Plasmodium falciparum* (Pf) is the primary malaria parasite species [59]. All healthy children and adults included in this study were afebrile and asymptomatic at the time of sample collection. Subclinical, asymptomatic Pf parasitemia was determined by either positive blood smear or positive Pf PCR. A blood smear slide was deemed negative when no parasites were seen after counting microscopic fields containing at least 200 leukocytes. Submicroscopic blood-stage infections were detected by a conventional nested PCR assay targeting 18S Pf–specific small subunit ribosomal RNA genes performed as previously described [60].

Healthy malaria-naïve US adult controls were enrolled in this study as an additional comparison group. The characteristics (age, sex, and Pf parasitemia status) of all study participants included in all assays for this study are summarized in Additional file 1: Table S1. This includes Kenyan children (*n* = 33), median age 5.1 years (range 1.2–9.8 years), 66.7% male, and 6 (18.2%) with subclinical asymptomatic Pf parasitemia; Kenyan adults (*n* = 30), median age 28.5 (range 18–35 years), 36.7% male, and none with asymptomatic Pf parasitemia; and US adults (*n* = 17), median age 28 years (range 24–69 years), 52.9% male, and none with asymptomatic Pf parasitemia.

### DNA methylation profiles

Monocytes were isolated from whole blood via negative selection with the RosetteSep Human Monocyte Enrichment Cocktail (Stemcell Technologies, 15,068). Infinium MethylationEPIC BeadChip arrays (Illumina, Inc.) were used to perform epigenome-wide DNA methylation analysis of isolated monocytes. This platform interrogates methylation at > 850,000 sites across the genome at single-nucleotide resolution. Genomic DNA from monocyte samples was bisulfite-converted (Zymo EZ DNA Methylation kit, D5001) and then hybridized to the array according to manufacturer’s instructions. Illumina methylation array data may show batch effects secondary to technical artifacts from different runs. Our data were collected over two different runs with four separate slides, which included 8 healthy children from western Kenya and the same 8 Kenyan children during a preceding episode of acute uncomplicated malaria (run 1, samples evenly dispersed over slides A and B); and 8 healthy adults from western Kenya and 8 malaria-naïve US adults (run 2, samples evenly dispersed over slides C and D). All quality control and normalization procedures were performed to include all four groups, and principal components analyses did not show significant association with slide for run 1 or run 2. This does not, however, exclude the potential for batch effects that may be present or that may affect only a portion of the genome.

#### Quality control and normalization

Data quality were assessed using the standard Bioconductor/R minfi pipeline (version 1.28.14) [61]. We filtered out probe sites with intensities below background (detection *P* values > 0.01; 4849 probe sites removed), probes at known SNPs, and probes on X and Y chromosomes. We normalized signal intensity using the functional normalization procedure [62], which accounts for between-array technical variation and background variation. After normalization and filtering, 809,761 probe sites remained for differential methylation analysis.

#### Differential methylation analysis

Normalized probe intensity values were used to calculate beta and M-values, where the beta value is the ratio of methylated probe intensity to the overall intensity and the M-value is log_2_ of the ratio of intensities of methylated vs. unmethylated probes [log_2_(β/(1-β))]. Differentially methylated positions (DMPs) were determined using a linear model from the limma package (v. 3.38.3) [63], with age group as the primary predictor variable, adjusting for sex and asymptomatic Pf infection status. Probe sites were considered differentially methylated if the absolute beta value difference was > 0.15. Statistical significance was set at *p* < 0.01 and false discovery rate (FDR)-adjusted *p* < 0.05. Differentially methylated regions (DMRs) were determined using the DMRcate package (v. 1.18.0) [64], which extracts and annotates DMRs using a kernel-smoothed estimate. Gaussian kernel bandwidth for smoothed-function estimation was set at lambda = 1000 nucleotides, with the scaling factor for bandwidth C = 2. Statistical significance was reported by the Stouffer combined *p* value, which is the transformation of the group of FDR-adjusted *p* values for individual CpG sites as DMR constituents [65]. Enrichment of probe sites in CpG islands, shores, or shelves was analyzed by comparing the proportions of age-associated hyper- or hypo-methylated probe sites in these regions to the MethylationEPIC assay coverage of probe sites in these regions.

#### Pathway enrichment analysis

KEGG pathway enrichment analysis was performed using the missMethyl package (v. 1.16.0) [66], which takes into account the bias in methylation arrays for gene set testing due to differing numbers of probes per gene. Input for enrichment analyses were the top differentially methylated probe sites with FDR-adjusted *p* values < 0.05 and absolute beta value difference > 0.15 (14,259 probe sites), tested against a background of all probe sites tested (809,761 probe sites).

#### Data availability

The DNA methylation data discussed in this publication have been deposited in NCBI’s Gene Expression Omnibus and are accessible through GEO Series accession number GSE157611 (https://www.ncbi.nlm.nih.gov/geo/query/acc.cgi?acc=GSE157611).

### Monocyte subset proportions by flow cytometry

Proportions of monocyte subsets were determined for 17 healthy children and 14 healthy adults from western Kenya and for 4 healthy malaria-naïve US adults, as previously described [35]. Briefly, peripheral blood mononuclear cells (PBMC) were separated by standard Ficoll-Hypaque density gradient centrifugation and cryopreserved. PBMC were gently thawed and resuspended at a concentration of 10^6^ cells/ml in RPMI-1640 (GIBCO). LIVE/DEAD Fixable Violet stain was used to assess cell viability (ThermoFisher Scientific). PBMC were directly stained for 20 min at 4 °C with Alexa Fluor 700–labeled anti-CD14 (clone 61D3; eBioscience) and APC-Cy 7–labeled anti-CD16 (clone 3G8; BioLegend). The stained cells were washed twice, fixed, and subjected to flow analysis on LSRII flow cytometer (BD Biosciences). FlowJo v8 software (Tree Star) was used for data analysis. The gating strategy put forth by the Nomenclature Committee of the International Union of Immunological Societies was used to determine proportions of monocyte subsets (classical CD14^++^CD16^−^, intermediate CD14^++^CD16^+^, and nonclassical CD14^+^CD16^++^) [67].

### Monocyte cytokine production

Monocyte cytokine production was analyzed using freshly isolated monocytes from 8 healthy Kenyan children, 10 healthy Kenyan adults, and 10 healthy US adults, as previously described [35]. Monocytes were isolated from whole blood via negative selection with the RosetteSep Human Monocyte Enrichment Cocktail (Stemcell Technologies, 15,068). Cells were suspended in culture medium (RPMI-1640 supplemented with 2 mM L-glutamine, 10% FBS, 10 mM HEPES, 1 mM sodium pyruvate, 4.5 g/l glucose, 1.5 g/l sodium bicarbonate, and 0.05 mM 2-ME) and placed in 96-well polypropylene plates at 5 × 10^4^ cells per well (concentration 5 × 10^5^ cells/ml). Cells were stimulated with 10 ng/ml LPS (Sigma-Aldrich) and 100 ng/ml Pam3CSK4 (P3C) (Invivogen) and compared with a media-alone control; each condition was performed in duplicate. Cells were cultured for 18 h at 37 °C, in 5% CO_2_, on an orbital shaker. Supernatants were harvested and stored at − 80 °C. A multiplex magnetic bead–based immunoassay was used to measure concentrations of IL-1β, IL-6, IL-8, IL-10, IL-12p70, and TNF (EMD Millipore) in the culture supernatants immediately after initial thawing.

### Targeted digital RNA sequencing

Monocyte inflammatory gene expression profiles were analyzed in samples from 6 Kenyan children, 7 Kenyan adults, and 4 US adults, as previously described [35]. Monocytes were isolated from cryopreserved PBMC via negative selection (Pan Monocyte Isolation Kit, Miltenyi Biotec, 130–096-537). Cells were lysed in RNAprotect Cell Reagent (Qiagen), and total RNA was prepared using the RNeasy kit (Qiagen) as per the manufacturer’s instructions. Total RNA integrity was assessed by an Agilent 2100 Bioanalyzer, and the RNA integrity number was calculated (≥8.5 for all samples). Targeted digital RNA sequencing was performed using a customized QIAseq Targeted RNA Panel (Human Inflammation and Immunity Transcriptome Panel, 508 genes) by the manufacturer (Qiagen).

#### Differential gene expression analysis

Raw QIAseq output in read counts per gene per sample were used as input for differential gene expression analysis using DESeq2 (v. 1.22.2) [68]. Significance for differential gene expression was set at FDR-adjusted *p* < 0.05. KEGG pathway enrichment analysis of differentially expressed genes was performed using [69].

### Statistics

Monocyte DNA methylation and gene expression statistical analyses were performed as described above using Bioconductor/R (v. 3.5.3). For the remainder of the assays, Mann-Whitney U test was used to compare continuous variables between groups. Kruskal-Wallis test was used to compare categorical variables (sex, Pf infection status) among healthy Kenyan children, Kenyan adults, and US adults. Differences were considered significant at *p* < 0.05. Graphs were constructed and statistical analyses were performed using Prism software (v. 8.2.1; GraphPad).

### Study approval

Informed consent was obtained from all participants or their guardians in the appropriate local language (English for US adults, Luo for Kenyan participants). Ethical approval was obtained from the Institutional Review Board of University Hospitals Cleveland Medical Center, Cleveland, Ohio, USA and from the Scientific and Ethical Review Unit (SERU) of the Kenya Medical Research Institute Ethical Review Committee.

## Supplementary Information

**Additional file 1 Table S1.** Study participant characteristics. **Figure S1.** Monocyte subset proportions in Kenyan adults and children.

**Additional file 2.** DMPs_Kenyan_child_vs_Kenyan_adult.csv.

**Additional file 3.** DMRs_Kenyan_child_vs_Kenyan_adult.csv.

**Additional file 4.** KEGG_meth_Kenyan_child_vs_Kenyan_adult.csv.

**Additional file 5.** DESeq2_Kenyan_child_vs_Kenyan_adult.csv.

**Additional file 6.** DMPs_Kenyan_child_vs_US_adult.csv.

**Additional file 7.** DMPs_Kenyan_adult_vs_US_adult.csv.

**Additional file 8.** KEGG_meth_Kenyan_adult_vs_US_adult.csv.

**Additional file 9.** DESeq2_Kenyan_adult_vs_US_adult.csv.

## Data Availability

The DNA methylation data discussed in this publication have been deposited in NCBI’s Gene Expression Omnibus and are accessible through GEO Series accession number GSE157611 (https://www.ncbi.nlm.nih.gov/geo/query/acc.cgi?acc=GSE157611). Supporting DNA methylation and gene expression data are included as supplementary material. All other supporting data are available from the corresponding author on reasonable request.

## Abbreviations

PRR: Pattern recognition receptor
DMP: Differentially methylated position
DMR: Differentially methylated region
SNP: Single nucleotide polymorphism
PCA: Principal components analysis
EWAS: Epigenome wide association study
KEGG: Kyoto Encyclopedia of Genes and Genomes
PBMC: Peripheral blood mononuclear cells
P3C: Pam3CSK4
