# Supplementary material for: Age-related differences in monocyte DNA methylation and immune function in healthy Kenyan adults and children
Source: Immun Ageing. 2021 Mar 8;18:11. doi: 10.1186/s12979-021-00223-2 (PMC7938546; doi:10.1186/s12979-021-00223-2)
Supplement: Supplementary file 1 — Additional file 1 Table S1. Study participant characteristics. Figure S1. Monocyte subset proportions in Kenyan adults and children. [file 12979_2021_223_MOESM1_ESM.docx]

**Table S1:** Characteristics of study participants

|  | **Kenyan Children** | **Kenyan Adults** | **US Adults** | ***p* value**^5^ |
| --- | --- | --- | --- | --- |
| **All participants** | | | |  |
| N | 33 | 30 | 17 |  |
| Age in years, median (range) | 5.1 (1.2, 9.8) | 28.5 (18, 35) | 28 (24, 69) |  |
| Male sex (%) | 22 (66.7) | 11 (36.7) | 9 (52.9) | 0.06 |
| Asymptomatic Pf infection^1^ (%) | 6 (18.2) | 0 | 0 | 0.01 |
| **DNA methylation arrays**^2^ | | | |  |
| N | 8 | 8 | 8 |  |
| Age in years, median (range) | 5.3 (3.6, 9.3) | 28.5 (26, 30) | 31 (25, 69) |  |
| Male sex (%) | 8 (100) | 5 (62.5) | 5 (62.5) | 0.15 |
| Asymptomatic Pf infection^1^ (%) | 4 (50) | 0 | 0 | 0.01 |
| **Monocyte subset proportions**^3^ | | | |  |
| N | 17 | 14 | 4 |  |
| Age in years, median (range) | 6.2 (1.2, 9.6) | 27.5 (18, 35) | 31 (26, 69) |  |
| Male sex (%) | 10 (58.8) | 3 (21.4) | 2 (50) | 0.11 |
| Asymptomatic Pf infection^1^ (%) | 2 (11.8) | 0 | 0 | 0.34 |
| **TLR responses**^2^ | | | |  |
| N | 8 | 10 | 10 |  |
| Age in years, median (range) | 6.8 (3.9, 9.8) | 28.5 (26, 30) | 27.5 (25, 36) |  |
| Male sex (%) | 7 (87.5) | 7 (70) | 4 (40) | 0.11 |
| Asymptomatic Pf infection^1^ (%) | 4 (50) | 0 | 0 | 0.004 |
| **Gene expression profiles**^4^ | | | |  |
| N | 6 | 7 | 4 |  |
| Age in years, median (range) | 4 (3.3, 4.9) | 29 (28, 33) | 30.5 (24, 47) |  |
| Male sex (%) | 3 (50) | 1 (14.3) | 1 (25) | 0.38 |
| Asymptomatic Pf infection^1^ (%) | 0 | 0 | 0 |  |

^1^ Asymptomatic Pf infection was defined as positive blood smear or Pf PCR without fever or other clinical malaria symptoms.

^2^ Monocytes were negatively selected from fresh venous blood samples.

^3^ Monocyte subset proportions were determined by flow cytometry of thawed cryopreserved PBMC samples.

^4^ Monocytes were negatively selected over magnetic column from thawed cryopreserved PBMC samples.

^5^ Kruskal-Wallis test was used to compare proportions with male sex and Pf infection among Kenyan children, Kenyan adults, and US adults.

Pf: *Plasmodium falciparum*; TLR: Toll like receptor; PBMC: peripheral blood mononuclear cells.

**Figure S1:** Monocyte subset proportions in Kenyan adults and children. (**a**) Gating strategy for proportions of circulating monocyte subsets. Doublets were excluded, and monocytes were gated based on forward- and side-scatter properties. Dead cells were excluded based on Fixable Violet staining. The 3 subsets were determined based on CD14 and CD16 expression (classical CD14^++^CD16^–^, intermediate CD14^++^CD16^+^, and nonclassical CD14^+^CD16^++^). (**b**) Proportions of all circulating monocytes that are classical, intermediate, and nonclassical for PBMC samples from Kenyan children (n = 17) and Kenyan adults (n = 14). Mann Whitney U test was used to compare the groups.
